# Selective Association Between Tetris Game Play and Visuospatial Working Memory: A Preliminary Investigation

**DOI:** 10.1002/acp.3339

**Published:** 2017-07-12

**Authors:** Alex Lau‐Zhu, Emily A. Holmes, Sally Butterfield, Joni Holmes

**Affiliations:** ^1^ Medical Research Council Cognition and Brain Sciences Unit Cambridge UK; ^2^ Social, Genetic and Developmental Psychiatry Centre, Institute of Psychiatry, Psychology and Neuroscience King's College London London UK; ^3^ Department of Clinical Neuroscience Karolinska Institutet Stockholm Sweden

## Abstract

Recent experimental and clinical research has suggested that Tetris game play can disrupt maladaptive forms of mental imagery because Tetris competes for limited cognitive resources within visuospatial working memory (WM) that contribute to imagery. Whether or not Tetris performance is selectively associated with visuospatial WM remains to be tested. In this study, young adults (N = 46) completed six standardized measures indexing verbal and non‐verbal reasoning, verbal and visuospatial short‐term memory, and verbal and visuospatial WM. They also played Tetris. Consistent with the hypothesis that visuospatial WM resources support Tetris game play, there was a significant moderate positive relationship between Tetris scores and visuospatial WM performance but no association with other cognitive ability measures. Findings suggest that Tetris game play involves both storage and processing resources within visuospatial WM. These preliminary results can inform interventions involving computer games to disrupt the development of maladaptive visual imagery, for example, intrusive memories of trauma. © 2017 The Authors. *Applied Cognitive Psychology* Published by John Wiley & Sons Ltd.

The popularity of video games among young people has sparked scientific and public interest in their impact on daily functioning and well‐being. Gaming has enormous applied potential in mental health, where healthcare services have traditionally struggled to engage young people (Patel, Flisher, Hetrick, & McGorry, 2007). Research in this area is still in its infancy, but one promising line of enquiry involves Tetris game play. Since its development, more than 30 years ago by Russian programmer and engineer Alexey Pajitnov, Tetris continues to be one of the most popular computer games worldwide (Fall, 2013). It has been used in research to promote cognitive enhancement (e.g. Belchior et al., 2013; Okagaki & Frensch, 1994), treat amblyopia or ‘lazy eye’ (Li et al., 2013), dampen vividness, and emotionality of autobiographical memories (Engelhard, van Uijen, & van den Hout, 2010), reduce cravings (Skorka‐Brown, Andrade, & May, 2014; Skorka‐Brown, Andrade, Whalley, & May, 2015), prevent intrusive memories of psychological trauma (Holmes, James, Coode‐Bate, & Deeprose, 2009; Iyadurai et al., 2017; James et al., 2015; James, Lau‐Zhu, Tickle, Horsch & Holmes, 2016b), and lessen mania‐related mental images (Davies, Malik, Pictet, Blackwell, & Holmes, 2012). Little is currently known about the cognitive mechanisms underpinning the benefits of Tetris, but one hypothesis emerging from the clinical psychology literature is that it selectively taxes *visuospatial* working memory (WM; Holmes et al., 2009; James et al., 2015). The aim of the current study is to test this assumption directly by investigating links between performance on the video game Tetris and visuospatial and verbal WM abilities in a group of young adults.

Tetris is a highly visuospatial game that involves the rotation and fitting together of visually presented blocks that differ in shape and colour. Evidence suggests that it activates regions of the brain associated with visuomotor processes, including sensory‐motor cortical dynamics (Rietschel et al., 2012) and occipitoparietal brain regions (Price, Paul, Schneider, & Siegle, 2013). Cognitive training studies have also shown benefits on visuospatial tasks following repeated practice on Tetris, with improvements observed in spatial ability (Okagaki & Frensch, 1994; Terlecki, Newcombe, & Little, 2008), mental rotation (De Lisi & Wolford, 2002; Moreau, 2013) and selective visual attention (Belchior et al., 2013; Green & Bavelier, 2003). Tetris game play can also lead to hypnagogic visual hallucinations that mix Tetris‐shape representations with other memories — the so call ‘Tetris effect’ (Kusse, Shaffii‐LE Bourdiec, Schrouff, Matarazzo, & Maquet, 2012; Stickgold, 2000).

The hypothesis that the beneficial effects of Tetris specifically involve demands on visuospatial WM (Holmes et al., 2009; James et al., 2015) comes from studies proposing that strategic Tetris game play can act as a ‘cognitive vaccine’ to minimize the development of maladaptive mental images across psychopathological states (James, Lau‐Zhu, Clark, et al., 2016a; Ji, Heyes, MacLeod, & Holmes, 2016). These include intrusive visual memories after psychological trauma (James et al., 2015), mania‐related visual images (Davies et al., 2012) and craving‐induced visual images of desire (Skorka‐Brown et al., 2014). Such beneficial effects of Tetris are hypothesised to occur when the visuospatial processing demands of game play compete for the same limited cognitive resources within WM (Baddeley, 2012; Baddeley & Hitch, 1974) that contribute to maintaining visual mental imagery in mind (Pearson, Naselaris, Holmes, & Kosslyn, 2015). The impact of the concurrent task is initially on weakening the mental imagery as held in WM — that is for brief periods of time (Baddeley & Andrade, 2000; Skorka‐Brown et al., 2014). However, this temporary disruption to the intensity of the image by WM interference is then thought to lead to it being permanently stored (Engelhard et al., 2010; Ranganath et al., 2005; van den Hout & Engelhard, 2012) in a weakened form in long‐term memory (LTM).

Supporting this idea, several lines of evidence suggest that WM is involved in the generation and maintenance of visual mental images. WM is a capacity‐limited cognitive system responsible for the temporary maintenance and processing of information (Baddeley, 2003; Unsworth & Engle, 2007) There are several models of WM, but the one most widely used in the study of mental imagery (e.g. Hitch, Brandimonte, & Walker, 1995; Baddeley & Andrade, 2000) is the multicomponent model developed by Baddeley and Hitch (1974) and later revised by (Baddeley, 2000). In this model, attentional control is provided by a domain‐general limited capacity central executive (e.g. Baddeley, 2000; Bayliss, Jarrold, Gunn, & Baddeley, 2003). Two specialized short‐term memory (STM) stores are responsible for the storage of verbal and visuospatial information (the phonological loop and visuospatial sketchpad, respectively), and a multimodal episodic buffer integrates information both within WM and across LTM systems (Baddeley, 2000, 2012). The capacities of the verbal and visuospatial stores are assessed by STM tasks involving the simple serial recall of information. These include measures such as digit span (verbal) and block recall (visuospatial). In addition to storage demands, tasks tapping the executive component also impose significant processing demands, and include measures such as backward digit recall (*reverse* serial order recall of verbal material) and Mr X (a visuospatial task involving spatial comparisons and simultaneous retention of serial order for spatial locations, see Figure 1). According to Baddeley's model, the processing demands of these tasks share variance and are therefore controlled by a domain‐general component (Bayliss et al., 2003). However, other competing accounts suggest that there are two independent domain‐specific components for verbal and visuospatial information, each capable of both storing and processing information (e.g. Friedman & Miyake, 2000; Jarvis & Gathercole, 2003; Miyake, Friedman, Rettinger, Shah, & Hegarty, 2001; Shah & Miyake, 1996). By these accounts, verbal WM (executive) tasks would not predict visuospatial abilities and vice versa.

**Figure 1:**
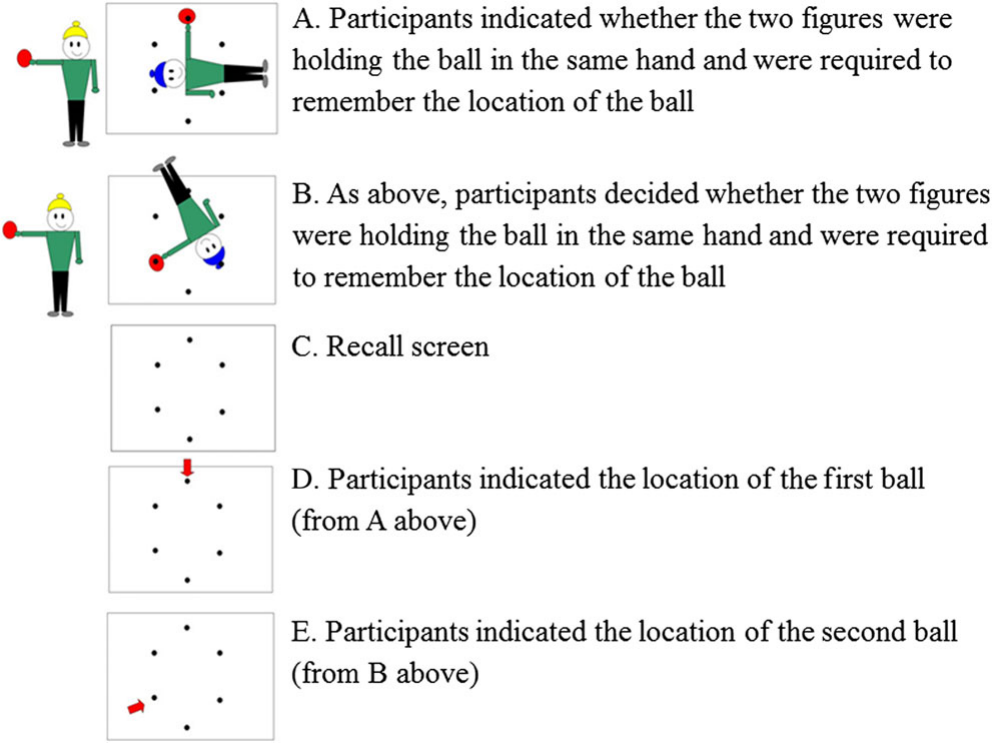
Screenshots of a single trial at a span level of two in the Mr X task (indexing visuospatial working memory) in sequence.

Performance on tasks measuring the visuospatial sketchpad are related to the ability to manipulate visuospatial images (e.g. Brandimonte, Hitch, & Bishop, 1992; Chambers & Reisberg, 1992; Hitch et al., 1995), and dual‐task studies have shown that concurrent WM tasks impair the generation of mental images in a modality‐specific manner. For example, Baddeley and Andrade (2000) reported that the vividness of visual mental images was reduced by a concurrent pattern‐tapping task designed to tax the visuospatial sketchpad but not by a concurrent counting‐aloud task designed to tax the phonological loop. The latter affected auditory mental images only. Similar effects have been reported for intrusive visual images using standardized visuospatial WM tasks (Deeprose, Zhang, Dejong, Dalgleish, & Holmes, 2012; Holmes, Brewin, & Hennessy, 2004; May, Andrade, Kavanagh, & Penfound, 2008) but less consistently using non‐visuospatial/verbal tasks (see Brewin, 2013 and James, Lau‐Zhu, Clark, et al. 2016a for a review on this topic).

A procedure involving Tetris game play has shown particular promise in reducing the number of intrusive memories of traumatic events. These memories are sensory‐based and affect‐laden comprising strong visual imagery (Bourne, Mackay, & Holmes, 2013; Holmes, Grey, & Young, 2005; Michael, Ehlers, Halligan, & Clark, 2005). Importantly, the Tetris procedure has been shown to reduce the number of subsequent intrusive memories of a traumatic film, even when it is played after a delay of half an hour (Deeprose et al., 2012; Holmes et al., 2009; Holmes, James, Kilford, & Deeprose, 2010) or up to 6 h later (Holmes et al., 2010), with recent successful translation to real‐life trauma (Iyadurai et al., 2017). These effects within the first few hours are consistent with the neuroscience of memory, which indicates that memory remains susceptible to interference after initial encoding (e.g. initial viewing of a trauma film) while the memory trace continues to consolidate into LTM with up to 6 h of delay (Nader, 2003; Walker, Brakefield, Hobson, & Stickgold, 2003). Emerging research suggests that during this post‐encoding period, newly acquired memory traces spontaneously reactivate or ‘replay’, promoting its gradual and persistent consolidation into LTM (Staresina, Alink, Kriegeskorte, & Henson, 2013). Tetris game play during the time window of memory consolidation may compete with shared WM resources involved in the spontaneous reactivation of trauma images in mind (Baddeley & Andrade, 2000), for example, rendering them less vivid and less emotional (Engelhard et al., 2010). This temporarily disrupted image in WM could then be stored and consolidated into LTM storage (Engelhard et al., 2010; Ranganath, Cohen, & Brozinsky, 2005; van den Hout & Engelhard, 2012).

Interestingly, the memory reconsolidation literature suggests that even established memories become labile again upon reactivation from LTM and need to re‐stabilize and reconsolidate to persist (Nader, 2003; Nader, Schafe, & Le Doux, 2000; Walker et al., 2003). Therefore, the effects of Tetris on intrusive memories could be observed after any length of post‐encoding delay in principle, as long as the memory is labile and thus amenable to interference, for example, as has been tested 24 h or more after encoding (James et al., 2015). Such effects are clinically relevant, as interventions may not always be readily deliverable immediately after trauma.

Together, these results suggest that visuospatial WM resources are needed to form visual mental images; thus, such images would be most optimally disrupted by tasks that compete with visuospatial WM resources. By this account, Tetris game play interferes with visual imagery (e.g., of trauma and cravings) by competing with both visuospatial sketchpad (storage) and central executive resources (processing). More specifically, the visuospatial sketchpad might provide support for holding the visuospatial shapes in the ‘mind's eye’ during Tetris game play, with the central executive supporting the mental rotation of the shapes to align them in their desired position. However, note that other authors in the clinical psychology literature have argued that Tetris may disrupt image‐based cognitions (the vividness of autobiographical memories) mainly by taxing the central executive (Engelhard et al., 2010; Gunter & Bodner, 2008; van den Hout & Engelhard, 2012).

The current study provides the first direct investigation into the association between Tetris game play and different aspects of WM in young people. A group of 16 to 18 year‐olds completed four standardized tests of WM, one each of verbal and visuospatial STM, and verbal and visuospatial WM. Participants also played Tetris, and completed assessments of verbal and non‐verbal reasoning to test the specificity of any associations with WM. The links between these measures were first explored using correlational analyses. On the basis of previously reported links between visual mental imagery and the visuospatial sketchpad, we expected Tetris game play scores to be positively correlated with visuospatial STM, but not with verbal STM. On the basis that Tetris involves the processing of information (e.g. mental rotation) in addition to storage, we also expected Tetris game scores to be positively correlated with all the WM tasks. According to domain‐general accounts of WM (e.g. Baddeley, 2000), links would be predicted between Tetris and both the verbal and visuospatial WM tasks, as the latter two are thought to share the central executive component; however, according to domain‐specific accounts of WM (e.g. Shah & Miyake, 1996), links would be predicted between Tetris and the visuospatial WM task only. The links between measures were also subsequently explored using principal component analyses to investigate the underlying factor structure. Tetris scores were expected to load on factors representing the visuospatial sketchpad and the central executive.

## Methods

### Participants

A total of 46 participants (7 men; mean age 16.98 years old, *SD* = 0.54) were recruited from four colleges in Southeast England. All but two participants were of British nationality and spoke English as their first language. The most common maternal highest educational level was secondary school (56.5%) followed by university degree (26.1%). Information about maternal education was not provided by 8.7% of the sample. For one participant, demographic information (except age) was not recorded owing to administration error. Participants provided written informed consent and were reimbursed for their participation. Ethical approval was obtained from the Cambridge University Psychology Research Ethics Committee [reference number Pre.2013.67].

### Tasks and measures

#### Tetris

A PC‐based version of Tetris set to ‘Marathon’ mode (Tetris Zone, version 1.2.1; Blue Planet Software, 2007) was played with the sound turned off (Laptop computer Dell Latitude E5530 with 15.6″ screen size). Different shaped blocks fell from the top to the bottom of the screen one at a time. The blocks were one of seven different colours and could be one of seven shapes. The aim of the game was to use the arrow keys to rotate and move each block as it was falling so that it formed a full horizontal line (without leaving any gaps) when it either reached the bottom or touched another block that had already fallen. Participants were rewarded points for each horizontal line completed, and all full lines of blocks were removed to make space for additional blocks to fall. The difficulty of the game increased as participants' performance improved (e.g., the blocks fell more quickly). The game ended when the blocks that had fallen were stacked to the top of the screen. To aid performance, the next three blocks due to fall after the one being played were presented on the right of the screen. Critically, participants were asked to ‘*try to work out in your mind's eye where best to place and rotate these blocks in order to make as many complete horizontal lines as you can and get the best score*’.

Participants were given 1 min to practice the game, and then 5 min to play; the game was restarted if it ended (game over) before the end of the 5‐min period. The computer program automatically generated a game play score at the end of each game, derived from the number of horizontal lines created and the level reached. Additional points were awarded if multiple lines were completed at the same time. Cumulative total scores were analysed. These were calculated by summing the total score for each game played during the 5 min (as in James et al., 2015). Participants also reported whether they have played Tetris before, and provided a yes/no answer.

#### General reasoning

The Peabody Picture Vocabulary Test (PPVT; Dunn & Dunn, 2007) was used to assess verbal reasoning abilities. Participants were asked to select from a choice of four the picture that best matched a word spoken aloud by a researcher. The Matrix Reasoning subtest of the Wechsler Abbreviated Scales of Intelligence Scale (WASI Matrix; Wechsler, 1999) was used to assess non‐verbal reasoning abilities. Participants were required to select a picture from a choice of five to complete a pattern consisting of abstract patterns and designs. Both tests increased in difficulty; The PPVT was discontinued when participants made eight errors in a block of 12 items, while the WASI Matrix was discontinued when participants made three consecutive errors. Standard scores were derived for the PPVT (*M* = 100, *SD* = 15) and *t* scores were derived for the WASI Matrix (*M* = 50, *SD* = 10).

#### Working memory

Participants completed four subtests from the Automated Working Memory Assessment (Alloway, 2007). The Automated Working Memory Assessment has demonstrated good internal validity (Alloway, Gathercole, Pickering, 2006), convergent validity with other assessment measures of WM (Alloway, Gathercole, Kirkwood & Elliot, 2008) and a clinical questionnaire of WM deficits (Alloway, Gathercole, Kirkwood & Elliot, 2009). For this study, verbal STM was indexed by digit recall, which required the immediate serial recall of increasingly long lists of verbally presented digits. Visuospatial STM was indexed by dot matrix that involved the immediate serial recall of a sequence of visually presented dots in a 4 × 4 matrix. Verbal WM was indexed by backward digit recall. For this task, participants recalled increasingly long lists of verbally presented digits in reverse order. Mr X was used to measure visuospatial WM. Participants were presented with pairs of cartoon figures (Mr X's, Figure 1). The task was to judge, for each pair, whether the two characters were holding the ball in the same hand or not. The Mr X on the right was presented in one of six possible rotated positions in each display, with the ball held at one of six compass locations. At the end of the sequence, the task was to recall the locations corresponding to the position of the ball held by the character on the right in each successive display. For all tasks, trials were presented in blocks; each block consisted of six trials. Sequences in the first block started at a span of two items and increased in length by one item in each subsequent block if participants scored four or more trials correct. A discontinue rule of more than two errors in any block was applied. Trials correct were scored to this point and converted to standard scores (*M* = 100, *SD* = 15). Test–retest reliability for the measures was: digit recall, .89; dot matrix, .85; backward digit recall, .86; Mr X, .84 (Alloway, 2007).

### Procedure

Participants completed the study individually with a researcher in a quiet area in school. The cognitive ability measures were completed first, followed by Tetris.

## Results

All measures were checked for normality and no univariate outliers (3 *SD*) were identified (Tabachnick & Fidell, 1996). Tetris scores were negatively skewed, so square root transformation was applied. Mean scores for each cognitive measure and Tetris score are shown in Table 1. An alpha level of .05 was used for all statistical tests.

**Table 1:** Descriptive statistics for all cognitive measures

Cognitive measure	*M*	*SD*	Range
General reasoning			
PPVT	100.41	11.32	82–130
WASI Matrix	43.93	8.99	20–59
STM and WM			
Digit Recall	95.90	10.24	74–126
Dot Matrix	97.46	16.94	70–138
Backward Digit Recall	98.75	12.08	73–126
Mr X	94.25	17.10	67–132
Tetris Game Play			
Total score^a^	65.32	28.72	20.59–139.18

*Note*: PPVT = Peabody Picture Vocabulary Test; WAIS = Wechsler Adult Intelligence Scale; STM = short‐term memory; WM = working memory;

a Scores were square root transformed.

### Correlational analysis

**Figure 2:**
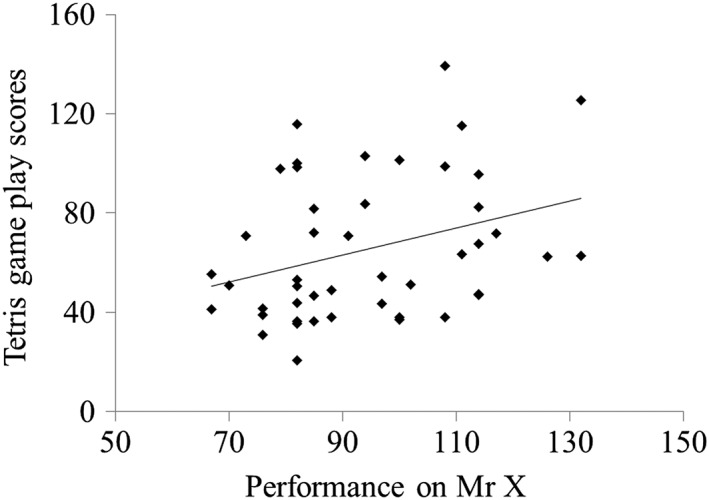
Scatterplot depicting the correlation between performance on Mr X and Tetris game play scores (*r* = .33, *p* = .028); Tetris game play scores were square root transformed; and standard performance scores were derived for Mr X (*M* = 100, *SD* = 15)

Correlations between the cognitive measures are shown in Table 2. There was a significant positive correlation between both IQ tests. The two tests of verbal aspects of WM were significantly related to one another, as were the two tests of visuospatial aspects of WM (*p* = .051). The two STM tasks were also related to one another. The IQ tests were associated with non‐verbal aspects of WM with significant correlations found between Matrix Reasoning and visuospatial STM, and between the PPVT task and visuospatial WM. Tetris scores were significantly and positively correlated with the visuospatial WM task (see Figure 2), but not with any of the other cognitive ability measures. While the substantial majority of the sample had previously played Tetris (84.8%), adding prior experience with Tetris as a covariate to the analyses did not impact on the overall patterns of association between Tetris game play and WM.

**Table 2:** Zero‐order correlations between measures of general reasoning, WM and Tetris game play score (*N* = 46)

Cognitive measure	1	2	3	4	5	6
General reasoning						
1. PPVT	—					
2. WAIS Matrix	.51**	—				
STM and WM						
3. Digit Recall	−.02	−.05	—			
4. Dot Matrix	−.13	.21	.16	—		
5. Backwards Digit recall	.14	.34*	.44**	.36*	—	
6. Mr X	.30*	.26	.26	.29	.28	—
Tetris Game Play						
7. Total score^a^	.16	.21	−.07	.13	.07	.33*

*Note*: PPVT = Peabody Picture Vocabulary Test; WAIS = Wechsler Adult Intelligence Scale; STM = Short‐term memory; WM = Working memory;

a Scores were square root transformed;

* *p* ≤ .05.

** *p* ≤ .01.

To seek for further evidence of the selective association between Tetris and Mr X, Meng's *z*‐tests (Meng, Rubin, & Rosenthan, 1992) were used to explore whether the size of the correlation between Tetris and Mr X was significantly bigger than the other relevant correlations with our current sample size. The size of the correlation between Tetris and Mr X was significantly bigger (one‐tailed) only to the size of the correlation between Tetris and digit recall — the measure of verbal STM (*Z* = 2.19, *p* = .014), consistent with the idea that Tetris does not demand the temporary storage of verbal information. Given that the correlation between Tetris and visuospatial WM is numerically the biggest (Table 2), the lack of significant effects on the other comparisons was likely due to insufficient power.

### Exploratory factor analysis

To investigate further, the relationship between Tetris scores and cognitive abilities, a principle components analysis was conducted on all measures. Varimax rotation was used to force differentiation between factors. Three factors emerged with eigen values greater than 1, explaining 32.49%, 20.88% and 14.80% of the variance, respectively. Factor loadings >.30 on the rotated factor matrix are shown in Table 3. The variables loading most highly on factor 1 were the two measures of general reasoning, with a smaller loading for the visuospatial WM task. This factor therefore represents general fluid abilities. The variables loading most highly on factor 2 were the measures of verbal aspects of WM, digit recall and backward digit recall, with smaller loadings for both the visuospatial WM tests; this factor is therefore predominantly associated with verbal WM. The tests of visuospatial aspects of WM and Tetris game scores loaded on factor 3 suggesting that they share variance.

**Table 3:** Exploratory factor analysis with measures of general reasoning, WM and Tetris game play scores (*N* = 46)

Descriptors	Factor		
	1	2	3
	General reasoning	Verbal WM	Visuospatial WM
PPVT	**0.912**		
WAIS matrix	**0.755**		
Digit recall		**0.830**	
Backwards digit recall		**0.773**	
Dot matrix		0.422	**0.659**
Mr X	0.335	0.306	**0.578**
Tetris game play score^a^			**0.764**

*Note*: Loadings are shown for three Varimax‐rotated factors and with loadings >.50 in bold; PPVT = Peabody Picture Vocabulary Test; WAIS = Wechsler Adult Intelligence Scale;

a Scores were square root transformed.

## Discussion

This study provides to our knowledge the first direct test of the assumption that Tetris game play is selectively associated with visuospatial WM. Performance on Tetris showed a moderate positive correlation with a standardized test of visuospatial WM, but not with measures of other cognitive abilities indexing verbal aspects of WM or general reasoning abilities. This pattern of associations was reflected in an exploratory factor analysis in which Tetris loaded on the same factor together with the measure of visuospatial STM and the measure of visuospatial WM. These findings are consistent with the hypothesis generated from the clinical psychology literature (James et al., 2015; Skorka‐Brown et al., 2014) that Tetris game play selectively taxes visuospatial WM resources. It is a hypothesis of great applied value to the development of mental health interventions. This assumption was previously derived indirectly from the observation that visual imagery is disrupted by both laboratory tasks designed to tax the visuospatial sketchpad and Tetris game play (e.g. Andrade, Kavanagh, & Baddeley, 1997; Deeprose et al., 2012; Holmes et al., 2004).

The factor structure derived from the four tests of WM included in the current study is consistent with domain‐specific models of WM that contain two separate components for verbal and visuospatial information, each responsible for both processing and storing information (e.g. Jarvis & Gathercole, 2003; Miyake et al., 2001; Shah & Miyake, 1996). Contrary to the multicomponent model (e.g. Baddeley, 2000), there was no evidence for separate STM stores or a single factor representing shared central executive resources across measures of both visuospatial and verbal WM. Instead, the data revealed two independent factors, with the verbal STM and verbal WM tests loading on one factor and the visuospatial STM and visuospatial WM tests loading on the other. Crucially, Tetris scores loaded on the same factor as the visuospatial memory tests indicating that, as predicted, they share variance. From a processes point of view, these data suggest Tetris game play selectively taxes both storage and processing resources within visuospatial WM. The lack of an association between Tetris scores and measures of IQ suggests that this relationship is not mediated by general fluid abilities and that there is something very specific about the common cognitive demands of visuospatial WM tasks and Tetris.

These data are consistent with the theory that Tetris protects against problematic visual imagery via modality‐specific effects (e.g. James et al., 2015; Skorka‐Brown et al., 2014). That is, Tetris game play may indeed disrupt visual imagery of traumatic memories or cravings by selectively competing with storage and processing resources in visuospatial WM, rather than competing with domain‐general executive resources as has been proposed by other theorists (Engelhard et al., 2010; Gunter & Bodner, 2008; van den Hout & Engelhard, 2012). Clearly this interpretation is only preliminary owing to the scale of the current study and the absence of any measures of the direct effects of Tetris on imagery. It does, however, pave the way for future investigations in this field.

Our study was preliminary only, but given the clear pattern of results, it is now worthwhile replicating the study in larger sample, with sufficient power to perform analyses such as direct statistical comparisons between the sizes of the different correlations and exploration of potential moderating factors including prior experience with Tetris. While the current data suggest specific links between WM and Tetris, it should be acknowledged that only four tests of WM were included. To extend and replicate these findings, future studies should include multiple assessments of the different aspects of WM to see converging results. The correlational nature of the study impedes definitive causal inferences. To add our understanding of the mechanisms involved, future studies should employ dual‐task methodologies to investigate the disruptive effects of secondary tasks designed to tap WM on Tetris game play.

To summarize, the present study investigated the association between Tetris game play scores and WM in a group of young people and found that higher Tetris scores were selectively associated with better visuospatial WM abilities but not with performance on other control measures of general cognitive ability or verbal WM. Such findings, that Tetris game play involves both storage and processing resources within visuospatial WM, can guide efforts to harness visuospatial video game play for cognitive enhancements and improved mental health. A promising area is the amelioration of intrusive mental images across emotional psychopathology (James, Lau‐Zhu, Clark, et al., 2016a). This approach may be particularly suitable for young people for whom gaming may represent an attractive intervention modality (Holmes et al. 2017; King, Greaves, Exeter, & Darzi, 2013). We lack therapists to deliver mental health treatments on the scale needed worldwide, and the application of science is essential to understand mechanisms behind psychological therapy innovation (Holmes, Craske, & Graybiel, 2014; Kazdin & Rabbitt, 2013). In the longer run, could efforts in cognitive science research transform everyday computer gaming into affordable, targeted and scalable therapeutic tools?
